# *Xanthomonas* Whole Genome Sequencing: Phylogenetics, Host Specificity and Beyond

**DOI:** 10.3389/fmicb.2016.01100

**Published:** 2016-07-15

**Authors:** Alice Boulanger, Laurent D. Noël

**Affiliations:** Laboratoire des Interactions Plantes-Microorganismes (LIPM), Université de Toulouse, Institut National de la Recherche Agronomique (INRA), Centre National de la Recherche Scientifique (CNRS), Université de Paul Sabatier (UPS)Castanet-Tolosan, France

**Keywords:** *Xanthomonas*, tomato, pepper, type III effector repertoire, host specificity

Crop diseases impact both yield and product quality and result in important health, economic, environmental, and societal issues worldwide (Scholthof, 2003; Strange and Scott, 2005). These diseases are caused by microorganisms like viruses, fungi, and bacteria. Among phytopathogenic bacteria, the genus *Xanthomonas* comprises 20 species which together infect more than 400 plant species, among which several important crops such as rice, cassava, cabbages, citrus, or tomato (Mansfield et al., 2012). In contrast, individual *Xanthomonas* species have a narrow host range usually restricted to a specific plant genus or species. The genetic and molecular bases for host range determination in *Xanthomonas* are essentially unknown at this date (Jacques et al., 2016): It likely involves bacterial factors enhancing the physiological adaptation to the plant environment (e.g., tropism, attachment, nutrition, degradation of plant compounds) or limiting the elicitation of plant immunity (Büttner and Bonas, 2010). Elicitors of plant immunity include both pathogen-associated molecular patterns (PAMP) and type III effectors (T3E) (Jones and Dangl, 2006). PAMP-triggered immunity (PTI) is the first layer of plant's innate immunity and strongly restricts host range for most bacteria. Key to pathogenicity and host range expansion is pathogen's capacity to suppress PTI. This is the prime function of most T3E known to date. T3E proteins are directly injected inside plant cells using a molecular syringe known as the type III secretion system. As a countermeasure, given T3E might specifically elicit potent immune responses known as effector-triggered immunity (ETI) in a set of plant species resulting in a reduced host range. It is thus key to determine pathogen T3E repertoires. Large scale sequencing of collections of phytopathogenic bacteria at the inter- and intra-specific level has revealed a large diversity of T3E repertoires leading to the definition of core vs. accessory T3E (Potnis et al., 2011; Bart et al., 2012; Roux et al., 2015).

The paper by Schwartz et al. (2015) perfectly illustrates the power of comparative genomics to understand pathogenicity and host specificity of this important genus of pathogens. The authors focussed on bacterial spot disease of tomato and pepper caused by three distinct *Xanthomonas* species [*gardneri* (*Xg*), *perforans* (*Xp*), and *euvesicatoria* (*Xe*)]. Draft genome sequences were determined for 67 strains sampled in the southeastern and midwestern United States between 1994 and 2013 and subjected to comparative analysis. Importantly, core genome analysis supported the recent reclassification of these strains in three distinct bacterial species (Jones et al., 2004) and evidenced further intraspecific organization of *Xp* strains. Identification, comparison, and analysis of T3E repertoires allowed the definition of surprisingly large core effectomes (24/29 T3E in *Xp*; 26/31 in *Xe*; 19/25 in *Xg*). Thirteen T3E are conserved among all three species. Identification of these core T3E and their sequence conservation should prove instrumental to design durable disease resistance strategies against bacterial spot disease in tomato and pepper. Such large interspecific core T3E repertoire might highlight the need for a future larger worldwide sampling of *Xanthomonas* infecting tomato and pepper. Beyond presence-absence of T3E, the conservation of T3E genes or proteins was also investigated in *Xe, Xp*, and *Xg* revealing the evolution and dynamics of these pathogenicity determinants. Overall, a lot more diversity is observed in T3E gene repertoires than in core genome analyses, suggesting a central role of horizontal gene transfer and distinct evolutionary forces applying to T3E genes. In *Xe*, the authors witnessed the evolution of T3E gene profiles over a 20 years period which could have suggested that such effect was driven by plant breeding and the introduction of novel tomato or pepper varieties. However, changes in prevalent bacterial species on tomato observed the last decade in different states (Florida, Ohio, Michigan) is not associated with strong changes in tomato cultivars used in commercial production fields (Potnis et al., 2015). Abiotic conditions could drive diversity and prevalent strains in diseased tomato fields. For instance, *Xg* is generally prevalent in cooler regions as observed for *Pseudomonas syringae* pv. *tomato*, another bacterial spot agent (Potnis et al., 2015). These two pathogens share several T3Es that are not present in other strains of *Xanthomonas* associated with bacterial spot. Horizontal gene transfer between different pathogens found in similar regions could be a strong source of diversity.

The second part of the analysis reports on host range determination in *Xp* by two T3E, AvrBsT and XopQ. Indeed, the authors demonstrate that the loss of *avrBsT* alone explains *Xp* pathogenicity on pepper while the double loss of *avrBsT* and *xopQ* renders *Xp* pathogenic on *Nicotiana benthamiana*. This result illustrates how plant immunity might restrict host range by ETI activation.

The manuscript by Schwartz and colleagues thus illustrates the potential of large-scale pathogenomics in understanding pathogen evolution and disease emergence and offers perspectives to design durable resistance against bacterial spot in tomato and pepper. Such approaches would be further reinforced by an improved sampling design, an increased number of sequenced genomes, and the use of complete genomes now accessible by SMRT sequencing (Pacific Biosciences). Such bioinformatics analyses should now be extended to pan genomes, plasmids, InDels, SNPs, recombinations, and use the pioneering approach developed by Guttman and colleagues (McCann et al., 2012) which followed evolutionary forces applied to individual *P. syringae* genes resulting in the identification of several novel PAMP elicitors.

## Author contributions

All authors listed, have made substantial, direct and intellectual contribution to the work, and approved it for publication.

## Funding

AB and LN are supported by the LABEX TULIP (ANR-10-LABX-41 and ANR-11-IDEX-0002-02) and the CROpTAL grant (ANR-14-CE19-0002-01).

### Conflict of interest statement

The authors declare that the research was conducted in the absence of any commercial or financial relationships that could be construed as a potential conflict of interest.
